# Relationships of gag-pol diversity between *Ty3/Gypsy *and *Retroviridae *LTR retroelements and the three kings hypothesis

**DOI:** 10.1186/1471-2148-8-276

**Published:** 2008-10-08

**Authors:** Carlos Llorens, Mario A Fares, Andres Moya

**Affiliations:** 1 Institut Cavanilles de Biodiversitat i Biología Evolutiva, Universitat de València, Polígono de la coma S/N, Paterna, Valencia, Spain; 2 Biotechvana, Parc Cientific, Universitat de Valencia, Paterna, Lab 16D Polígono de la coma S/N, Paterna, Valencia, Spain; 3 Department of Genetics, University of Dublín, Trinity Collage Dublín, Dublín 2, Ireland; 4 CIBER de Epidemiología y Sal ud Pública (CIBERESP), Spain

## Abstract

**Background:**

The origin of vertebrate retroviruses (*Retroviridae*) is yet to be thoroughly investigated, but due to their similarity and identical gag-pol (and env) genome structure, it is accepted that they evolve from *Ty3/Gypsy *LTR retroelements the retrotransposons and retroviruses of plants, fungi and animals. These 2 groups of LTR retroelements code for 3 proteins rarely studied due to the high variability – gag polyprotein, protease and GPY/F module. In relation to 3 previously proposed *Retroviridae *classes I, II and II, investigation of the above proteins conclusively uncovers important insights regarding the ancient history of *Ty3/Gypsy *and *Retroviridae *LTR retroelements.

**Results:**

We performed a comprehensive study of 120 non-redundant *Ty3/Gypsy *and *Retroviridae *LTR retroelements. Phylogenetic reconstruction inferred based on the concatenated analysis of the gag and pol polyproteins shows a robust phylogenetic signal regarding the clustering of OTUs. Evaluation of gag and pol polyproteins separately yields discordant information. While pol signal supports the traditional perspective (2 monophyletic groups), gag polyprotein describes an alternative scenario where each *Retroviridae *class can be distantly related with one or more *Ty3/Gypsy *lineages. We investigated more in depth this evidence through comparative analyses performed based on the gag polyprotein, the protease and the GPY/F module. Our results indicate that contrary to the traditional monophyletic view of the origin of vertebrate retroviruses, the *Retroviridae *class I is a molecular fossil, preserving features that were probably predominant among *Ty3/Gypsy *ancestors predating the split of plants, fungi and animals. In contrast, classes II and III maintain other phenotypes that emerged more recently during *Ty3/Gypsy *evolution.

**Conclusion:**

The 3 *Retroviridae *classes I, II and III exhibit phenotypic differences that delineate a network never before reported between *Ty3/Gypsy *and *Retroviridae *LTR retroelements. This new scenario reveals how the diversity of vertebrate retroviruses is polyphyletically recurrent into the *Ty3/Gypsy *evolution, i.e. older than previously thought. The simplest hypothesis to explain this finding is that classes I, II and III trace back to at least 3 *Ty3/Gypsy *ancestors that emerged at different evolutionary times prior to protostomes-deuterostomes divergence. We have called this "the three kings hypothesis" concerning the origin of vertebrate retroviruses.

## Background

Attention was first drawn to the *Retroviridae *when HTLV-1 was characterized as pathogenic in humans [1,2]. They further increased in significance with the discovery of HIV-1, the retrovirus responsible for AIDS in humans [3,4]. These 2 retroviruses represent only a small part of *Retroviridae *diversity, which can be divided in seven genera; *Alpha-, Beta-, Gamma-, Delta-, Epsilon-, Spumaretroviridae *and *Lentiviridae *(according to ICTV classification [5]). Based on their strategy of transmission, the *Retroviridae *can also be classified as endogenous retroviruses when they enter the germ lines of hosts and are vertically transmitted; or as exogenous retroviruses, when they can be transmitted horizontally from one host into another via infection. Most recent trends in *Retroviridae *taxonomy [6-10] group endogenous and exogenousretroviruses into 3 major classes designated as I, II and III. Both classifications are complementary as class I comprises gamma- and epsilonretroviruses; class II includes lentiviruses, delta-, alpha- and betaretroviruses; and class III groups spumaretroviruses with ERV-L retroelements. The ancient history of the *Retroviridae *is yet to be thoroughly investigated, but due to their similarity and identical gag-pol (and env) genome structure, it is usually assumed that they evolve from the *Ty3/Gypsy *LTR retroelements of plants, fungi and animals [11]. The traditional view suggested by pol polyprotein domains such as the RT [12-14], RNAse H [14,15], and INT [14,16] used to resolve the phylogeny, delineates a common *Ty3/Gypsy *origin for all vertebrate retroviruses. Nevertheless little is known about this scenario because RT, RNAse H and INT analyses appear unable of agreeing on a precise well-supported *Ty3/Gypsy *root for the *Retroviridae*. In an attempt to bring light on this topic, we investigated 120 non-redundant *Ty3/Gypsy *and *Retroviridae *taxa based on the phylogenetic analysis of both gag and pol polyproteins. Our results revealed conflicting phylogenetic signals between these 2 polyproteins. From that point, we aimed to investigate more in depth this evidence through comparative analyses performed based on 3 independent proteins rarely considered by prior studies due to their variability – the gag polyprotein, the PR and the GPY/F module. Our study reveals taxonomic differences among the 3 *Retroviridae *classes, and an evolutionary network that distantly relates each class with one or more *Ty3/Gypsy *lineages. This observation appears to be at odds with the traditional monophyletic view suggested by prior approaches to determining the origin of vertebrate retroviruses, but requires further study. In light of this new perspective, we introduce here a new hypothesis for debate and further evaluation. Our hypothesis argues that classes I, II and III probably trace back to at least 3 independent *Ty3/Gypsy *ancestors. We call this the *three kings hypothesis*.

## Results

### Consistency of lineages but conflicting phylogenetic signals between gag and pol polyproteins in the *Ty3/Gypsy *and *Retroviridae *evolutionary history

**Figure 1:**
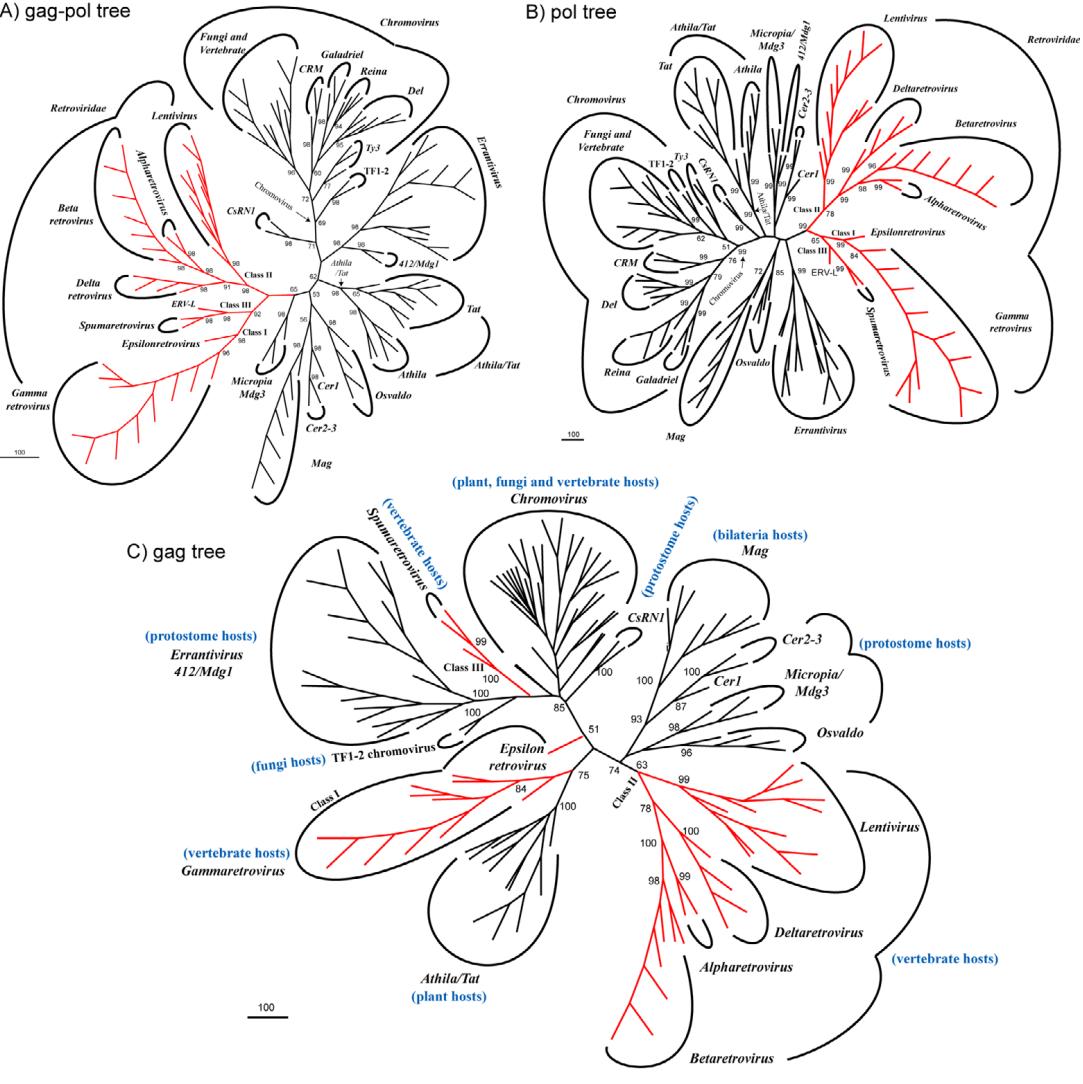
**Phylogenetic analyses**. A)*Ty3/Gypsy *and *Retroviridae *phylogeny inferred based on the concatenated analysis of both gag and pol polyproteins. This tree is robust as gag and pol signals complement and correct each other. It also supports with significant bootstrap values the 2 groups of LTR retroelements and all their accepted lineages (clades, genera and classes). An extended version of this tree facilitating names, lineages, hosts, and Genbank accessions of all retroelement taxa used is provided as the Additional file 1 accompanying this paper (see the Section "Sequences and databases" in Methods). Decomposition of gag-pol tree and analysis of its two components separately, reveals similar phylogenetic signal but conflicting evolutionary perspectives. B) The phylogenetic signal of the pol polyprotein is robust and therefore responsible for the current known taxonomy and classification of *Ty3/Gypsy *and *Retroviridae *LTR retroelements into lineages. C) The gag signal supports the clades, genera and classes described in each group, but does not supports the 2 groups. Gag tree outlines an alternative scenario that may relate each *Retroviridae *class with one or more *Ty3/Gypsy *lineages.

In a prior study [17], we used the inferred phylogenetic reconstruction of *Ty3/Gypsy *and *Retroviridae *LTR retroelements based on both gag and polpolyproteins as the criterion to create phylogenetically informative HMM profiles [18]. Figure 1A shows a radial version of this tree, which clearly supports the usually accepted monophyly of the *Ty3/Gypsy *and *Retroviridae *groups and all their assumed lineages (clades, genera and classes) [5-14,19-25]. This view of the origin of *Retroviridae *indicates that these retroviruses had a common origin, e.g. a *Ty3/Gypsy *LTR retrotransposon (for more information in this topic, see [11] and references therein). Interestingly, inferred gag-pol tree suggests a putative *Retroviridae *root in the *Ty3/Gypsy *evolutionary history, which according to this new analysis, is close to *Micropia/Mdg3 *clade [14] and other *Ty3/Gypsy *lineages described in bilateria genomes. This perspective suggests that the first *Retroviridae *ancestor emerged before or during the split between protostomes and deuterostomes together with several *Ty3/Gypsy *lineages, which apparently have distant counterparts (*Athila *and *Tat *clades [19,20]) in the genomes of plants. Taking into account that the *Retroviridae *are true viruses capable of escaping their hosts, this scenario might also be traced back to an ancient horizontal transference from protostomes to vertebrates and the colonization of the vertebrate genomes by these viral agents from that point on. However these two alternatives, whilst equally exciting perspectives, should be re-evaluated based on the separate analysis of gag and pol polyproteins. The phylogenetic analysis of the pol polyprotein (Figure 1B) is consistent with gag-pol tree, due to the grouping of the taxa into clusters. In fact, the bootstrap robustness of the different clades and genera reported by gag-pol tree comes from the strong pol phylogenetic signal. This means that the pol signal is the essential analytical substrate responsible for the current view on the evolutionary history and taxonomy of *Ty3/Gypsy *and *Retroviridae *LTR retroelements. However, the pol signal does not support the *Retroviridae *root suggested by the gag-pol tree, and does not reveal a well-supported alternative link between the *Ty3/Gypsy *and *Retroviridae *groups. Pol tree is consistent with gag-pol tree in to delineate a scenario of emergence for vertebrate retroviruses preceding the protostomes-deuterostomes split. However, the root suggested by pol tree falls close to errantiviruses the canonical *Ty3/Gypsy *retroviruses of flies [26-28]. Indirectly, this indicates that whatever the relationship between *Micropia/Mgd3 *clade and the *Retroviridae*, the relationship depends on the gag polyprotein. Consistent with this, the independent phylogenetic analysis of the gag polyprotein (Figure 1C) groups the *Retroviridae *class II with *Micropia/Mdg3 *clade and other *Ty3/Gypsy *lineages described in bilateria genomes. The gag phylogeny also reveals how the *Ty3/Gypsy *origin of vertebrate retroviruses is anything but straightforward. This tree also clusters gammaretroviruses (class I) with the *Athila/Tat *clades of plants, and suggests proximity between the *Retroviridae *class III and errantiviruses, and other *Ty3/Gypsy *lineages. In other words, the gag signal fails to support the monophyly of the two *Ty3/Gypsy *or *Retroviridae *groups and suggests an alternative scenario. That is, based on gag and depending on the class, it follows that the *Retroviridae *code for different gags, each having one or more distant counterparts among *Ty3/Gypsy *LTR retroelements.

### Retroviridae differentiation into classes outlines phenotypic differences in the gag polyprotein that distantly relate each class with one or more *Ty3/Gypsy *lineages

**Figure 2:**
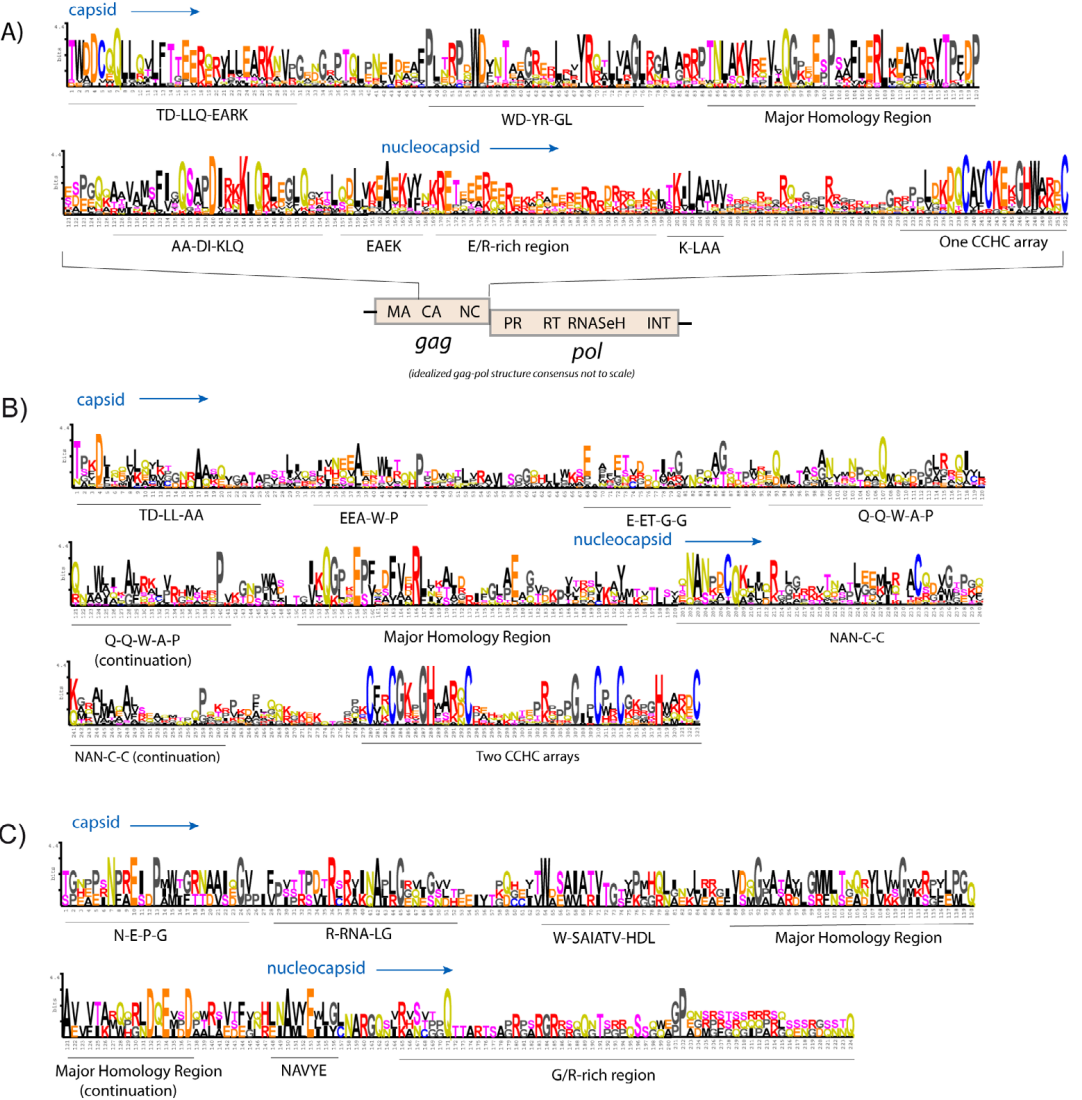
**Phenotypic capsid-nucleocapsid differences of the gag polyprotein based on the three classes**. *Retroviridae *differentiation into 3 previously proposed classes suggests how vertebrate retroviruses code for 3 different gag polyproteins, based on the CA-NC region. A) Sequence logo describing the CA-NC region coded by all gamma- and epsilonretroviruses (class I) used in this study. Class I gag exhibits several features (underlined in the Figure) the presence of a single CCHC array at NC being the most prominent. B) Sequence logo describing the class II CA-NC region was built on an alignment including lentiviral (HIV-1, HIV-2, SIVMAC, VMV, SA-OMVV and CAEV), betaretroviral (MPMV, SERV and SRV-1), alpharetroviral (LPDV and RSV), and deltaretroviral (HTLV-1, HTLV-2 and BLV) sequences. Class II gag amino acidic architecture is similar but displays important differences from that of class I. Note, for instance, how the C-terminus of class II gag is based on a trait we call "NAN-C-C-KA-P" followed by 2 CCHC arrays at NC. C) Sequence logo constructed based on all class III gags used. Class III gag has a CA trait extremely dissimilar from those of classes I and II. On the other hand, class III NC equivalent trait is rich on residues having similar physiochemical properties to those displayed in class I, but have no CCHC arrays.

**Figure 3:**
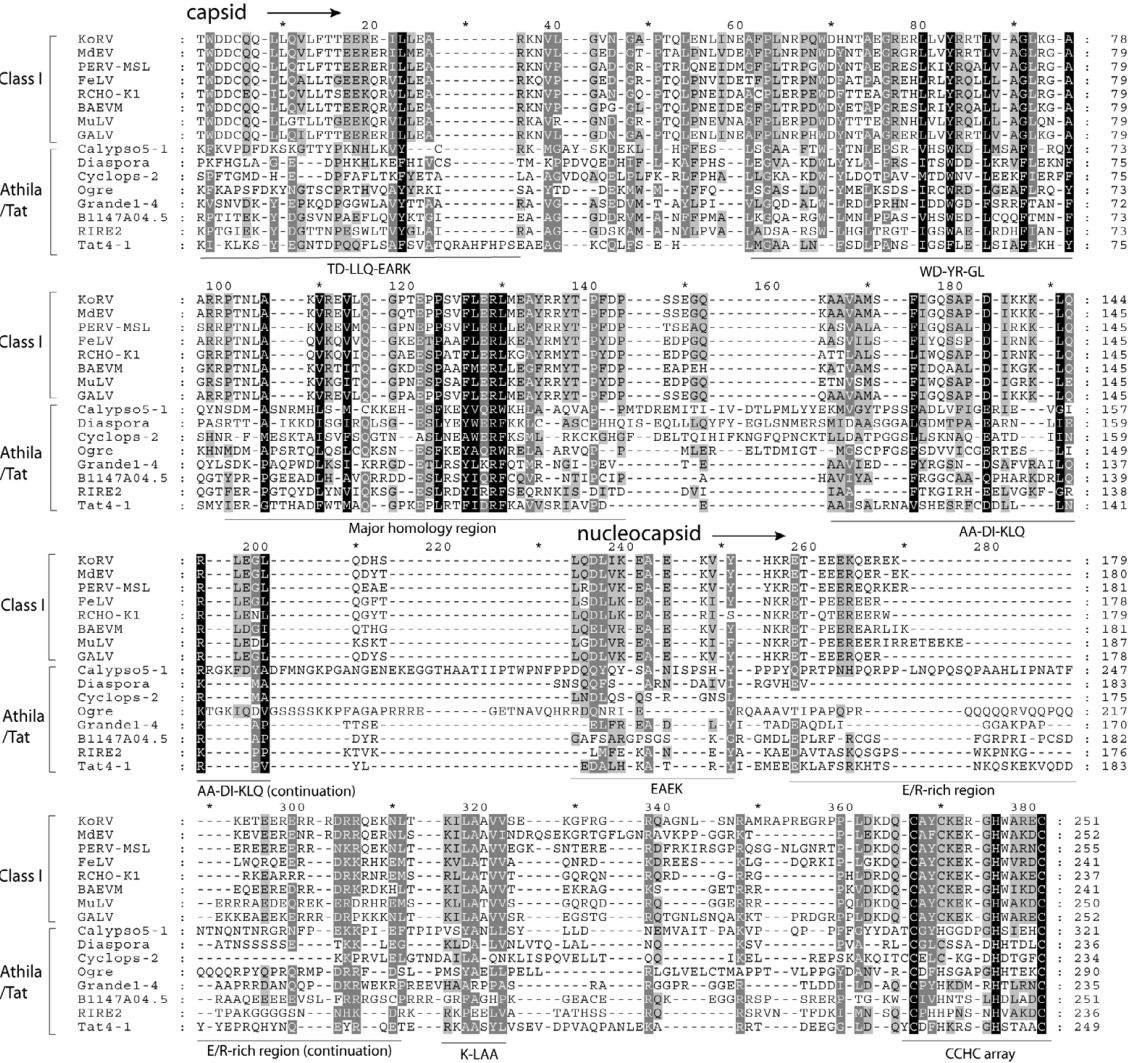
**Gag comparison between class I and *Athila/Tat *LTR retroelements of plants**. The *Retroviridae *differentiation into the 3 classes reveals how based on the CA-NC region, class I gammaretroviruses and *Athila/Tat *LTR retroelements of plants are more similar than previously supposed. Among others features in common (underlined and named following the nomenclature of Figure 2), both Athila/Tat and class I gags are characterized by the presence of a single CCHC array at NC. Note, however, how Tat NCs exhibit a CHHC motif substituting the canonical CCHC array.

**Figure 4:**
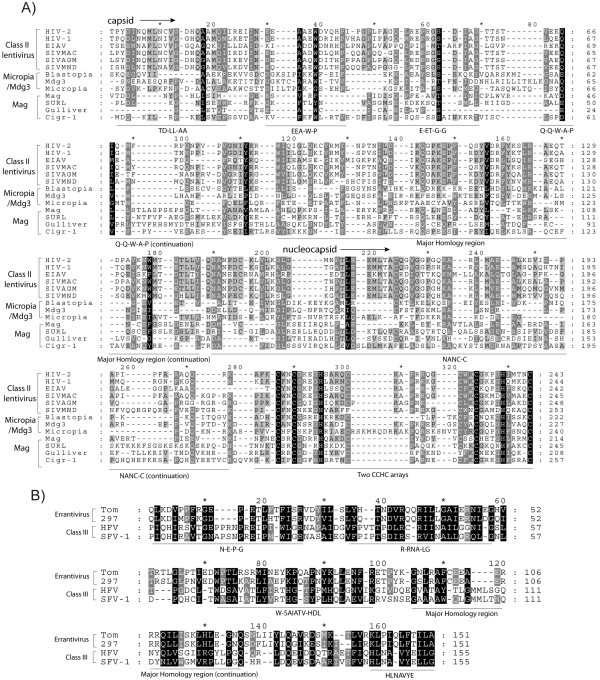
**Gag comparisons between classes II and III and bilateria *Ty3/Gypsy *LTR retroelements**. Based on the CA-NC region, classes II and III have counterparts among the *Ty3/Gypsy *LTR retroelements described in bilateria organisms. A) Multiple alignments of *Micropia/Mdg3 *and *Mag *clades and class II lentiviruses (the phenotypic features in common following the nomenclature of Figure 2 are underlined and named). Note the particular NC similarity based on the common presence of two CCHC arrays plus an additional trait displaying the trace of a NAN-C-C motif. B) Multiple alignment showing domain similarity between spumaretroviruses (class III) and several *Ty3/Gypsy *errantiviruses based on gag. Neither errantiviral nor spumaretroviral gags have CCHC arrays at their C-terminus.

Phylogenetic analyses performed based on gag are rarely reported, due to the fast rate of evolution of this polyprotein. However, the alignment from which we inferred the gag tree was manually constructed and its accuracy tested by comparative analyses. We contrasted all gag sequences with each other using the NCBI BLAST search [29] available at GyDB. Comparisons revealed that gag sequences belonging to a *Ty3/Gypsy or Retroviridae *clade, genus or class are usually more similar to their lineage counterparts than to other gag sequences (data not shown). This analysis also revealed a core of similarity that is common to all Ty3/Gypsy and Retroviridae gags. This core spans the CA-NC region and its most conserved traits appear to be the MHR at CA [30], and the zinc finger Cys-X2-Cys-X4-His-X4-Cys (CCHC) array at NC [31]. Evaluation of this core shows that the *Retroviridae *code for 3 different types of gag, each exhibiting a particular amino acidic architecture phenotype that depends on the class differentiation. While the 2 *Retroviridae *classes I and II appear to be related according to BLAST analyses (data not shown), they present greater divergence based on several phenotypic features preserved depending on the class (Figure 2A and 2B). Class III is extremely dissimilar to classes I and II based on gag, but preserves several features at the C-terminus that might be distantly related or equivalent to those of class I (Figure 2A and 2C). The most prominent, but obviously not unique, difference between the 3 classes is the variability in the number of CCHC arrays at NC. Class I NCs usually show one CCHC array, class II NCs exhibit two, and class III gags have no CCHC arrays at their C-terminus. BLAST analyses also revealed how the *Ty3/Gypsy *lineages related to classes I and II by gag tree, display greater similarity to different *Retroviridae *taxa belonging to these 2 classes than to other *Ty3/Gypsy *lineages. As an example, Tables 1 and 2 summarize the top similarity hits obtained from 4 comparisons conducted using 2 Micropia/Mdg3 and 2 Tatgag sequences as queries. All BLAST analyses were supported by additional sequence comparisons between the different gag queries and the collection of HMM profiles, available at GyDB via the HMM server (data not show). Additionally, we provide qualitative evidence of this relationship through alignment comparisons. Figure 3 shows a multiple alignment revealing domain similarity between gammaretroviruses (i.e. class I) and the *Athila *and *Tat *clades of plants. Figure 4A demonstrates that *Micropia/Mdg3 *clade and other bilateria *Ty3/Gypsy *lineages, such as the *Mag *clade, code for gags following similar CA-NC architecture to class II lentiviral gags. Gag relationship similarities between class III and other *Ty3/Gypsy *or *Retroviridae *lineages are not supported by BLAST analyses. However, Figure 4B shows a multiple alignment between spumaretroviruses and errantiviruses, which according to the qualitative domain similarity merits further attention.

**Table 1:** Hits of BLASTp similarity between Micropia/Mdg3 and other Ty3/Gypsy and Retroviridae gags

Query: Micropia gag			Query: Mdg3 gag		
Element	Score	E-value	Element	Score	E-value

*EIAV	51.2	1e-08	*HIV-2	45.4	9e-07
*SA-OMVV	43.1	3e-06	*SIVMAC	44.7	2e-06
Beetle1	42.4	5e-06	*SIVMND	43.9	3e-06
*HIV-2	42.0	7e-06	*HIV-1	42.4	8e-06
Pyggy	40.0	3e-05	*HTLV-2	38.9	9e-05
*FIV	40.0	3e-05	*STcLV2PP1664	38.1	1e-04
Real	38.5	7e-05	Legolas	37.0	3e-04
Skippy	38.1	1e-04	*EIAV	36.6	4e-04
*CAEV	38.1	1e-04	*FIV	36.2	6e-04
*SIVMAC	37.7	1e-04	*BIV	35.8	7e-04
SURL	37.4	2e-04			
Cer4	37.4	2e-04			
*RCHO-K1	36.2	4e-04			

We only summarize the most significant (top) hits of similarity obtained with each search. Retroviridae gags belonging to class II are indicated with asterisks

**Table 2:** Hits of BLASTp similarity between Tat and other Ty3/Gypsy and Retroviridae gags

Query: Retrosor1 gag			Query: Tat4-1 gag		
Element	Score	E-value	Element	Score	E-value

Diaspora	50.4	5e-08	*KoRV	34.3	0.003
Calypso5-1	42.4	1e-05	*GALV	33.5	0.005
Ulysses	40.0	6e-05	*HERV-K10	32.0	0.015
*GALV	39.7	8e-05	*PERV-MSL	30.8	0.033
*KoRV	39.3	1e-04	*SRV-1	29.6	0.074
*MdEV	38.1	2e-04	*MPMV	29.6	0.074
*PERV-MSL	37.7	3e-04	*MuLV	29.6	0.074
Cer3	36.2	0.001	*SERV	29.3	0.097
Cyclops-2	36.2	0.001	*JSRV	28.9	0.13
*MuLV	34.3	0.003			
Sushi-ichi	32.3	0.013			
*BAEVM	28.9	0.15			

We only summarize the most significant (top) hits of similarity obtained with each search. Retroviridae gags belonging to class I are indicated with asterisks.

Comparative analyses confirm phenotypic features in the gag polyprotein that distantly relate each *Retroviridae *class with one or more of the *Ty3/Gypsy *lineages evaluated. The similarity spans the CA-NC core and the most prominent feature in common is the variability in the number of CCHC arrays per NC. With very few exceptions, the *Athila/Tat *elements of plants usually code for NCs exhibiting one CCHC array, *Micropia/Mdg3 *and *Mag *elements code for NCs usually exhibiting 2 arrays (except *Mag *elements of *C.elegans*), and errantiviral gags have not CCHC arrays at their C-terminus. This indicates that the number of CCHC arrays per NC is evolutionarily preserved depending on the *Ty3/Gypsy *lineage and the *Retroviridae *class, and that this phenotype is an excellent indicator of taxonomy and evolution. For simplicity's sake, we do not discuss all *Ty3/Gypsy *cases. We discuss but one example, the most interesting instance of using this indicator – the chromodomain-containing *Ty3/Gypsy *LTR retrotransposons [14] called chromoviruses [13]. Chromoviruses are the most ancient branch of *Ty3/Gypsy *LTR retroelements as they have been described in the genomes of plants, fungi and vertebrates (for a more extensive information about chromoviruses, see [23,32,33]). It noteworthy that all *Ty3/Gypsy *LTR retroelements of plants can be divided in 2 major branches – chromoviruses and *Athila/Tat *– and that chromoviruses appear to be the only branch of *Ty3/Gypsy *LTR retroelements capable of colonizing the genomes of fungi. A prior study [30] reported that this branch of *Ty3/Gypsy *LTR retroelements displays similarity (we confirm) to gammaretroviruses based on CA-NC. However, we have also found how that chromoviruses show similarities to class II in addition to a number of *Ty3/Gypsy *lineages (for this reason chromoviruses fall at an intermediate position in the gag phylogeny). With rare exceptions, NCs coded by chromoviruses usually bear one CCHC array (data not shown). In contrast, the different *Ty3/Gypsy *lineages described in bilateria organisms show greater variability in the number of CCHC arrays at NC thantheir *Ty3/Gypsy *counterparts of plants and fungi (i.e. chromoviruses and the *Athila/Tat *branch). Gag evidence thus relates class I to the most likely CA-NC phenotype of *Ty3/Gypsy *ancestors predating the split between plants and the ophistokonts (fungi and animals) and classes II and III with other CA-NC phenotypes, more frequently observed among the *Ty3/Gypsy *LTR retroelements of protostomes and deuterostomes.

### Retroviridae differentiation into classes reveals three protease isoforms based on flap motif polymorphisms, which are common to *Ty3/Gypsy *and *Retroviridae *LTR retroelements

Through phylogenetic analyses, we have shown that the pol signal is primarily responsible for the branching of *Ty3/Gypsy *and *Retroviridae *LTR retroelements in 2 monophyletic groups. That is the usual evolutionary perspective based on the RT and other pol polyprotein domains. We have also shown that gag signal discloses an alternative scenario wherein each *Retroviridae *class can be related to one or more *Ty3/Gypsy *lineages. An in-depth examination of gag diversity through comparative analyses has revealed the phenotypic variations involved in this differential similarity. Gag evidence is thus well supported. An interesting question is whether this evidence should be considered a convergence due to the fast rate of evolution of the gag polyprotein, or if it is due to an ancient divergence. Certainly, the most robust components of the pol polyprotein – the RT, RNAse H and INT – usually support the traditional perspective originally delineated by RT analyses [12]. However, the strong signal from these 3 proteins disguises the particular perspective provided by another pol protein domain – the PR. Non-redundant studies focusing on Ty3/Gypsy and Retroviridae PRs are rarely reported as this enzyme presents identical analytical difficulty to gag due to its fast rate of evolution. Despite this it is well known that LTR retroelement PRs in general are aspartic peptidases belonging to clan AA (following MEROPS Database classification [34]). Within clan AA, Retroviridae PRs are divided into 2 protein families, retropepsins (family A2) and spumaretropepsins (family A9). Family A2 groups all PRs coded by classes I and II and family A9 collects the PRs coded by spumaretroviruses (class III). Such a classification keeps going because retropepsins and spumaretropepsins are strongly dissimilar each other and do not group on a single branch in any analysis (data not shown). On the other hand, Ty3/Gypsy PRs are extremely variable and little is known about them. MEROPS Database at least classifies many Ty3/Gypsy examples within family A2 because these PRs display great similarity to retropepsins. However, not all Ty3/Gypsy PR are similar to retropepsins as not all Retroviridae PRs are retropepsins. Because no study evaluates the relationships between Ty3/Gypsy and Retroviridae PRs, we investigated this topic, taking into consideration the differentiation of the 2 groups of LTR retroelements into lineages. It is worth remembering that while gag and pol signals are in disagreement over the taxonomical groups, they do support the differentiation into clades, genera and classes of *Ty3/Gypsy *and *Retroviridae *LTR retroelements.

**Figure 5:**
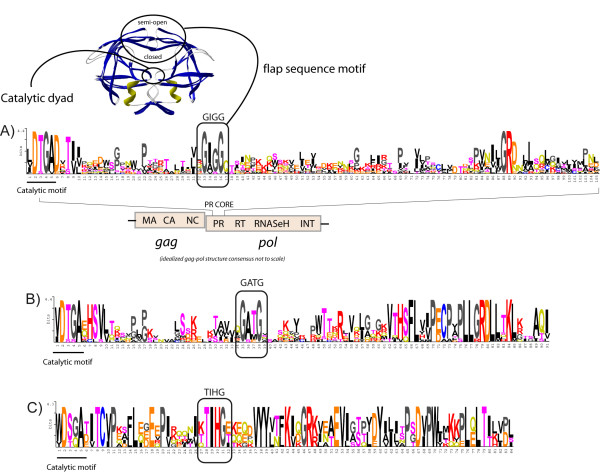
**Retroviridae protease isoforms**. Retroviridae PRs dimerize in their active form and each lobe of this enzyme usually has a structural flap (the two β-hairpin loops enclosed in a circle covering the catalytic DT/SG dyad). *Retroviridae *differentiation into the 3 classes reveals 3 different isoforms of the same enzyme, each exhibiting a particular flap motif. A) Sequence logo describing class II PRs, the flap correspondence on sequence in this PR is a GIGG amino acid motif included in a box. B) Sequence logo describing class I PRs; this variant preserves a GATG motif at the same flap sequence position. C) Sequence logo built based on class III PRs revealing a TIHG motif in this position. To improve the visualization on amino acidic architecture, we have used the HFV and SFV-1 sequences (see methods) plus FFV (Genbank accession CAA70075), FSV (AAC58531), SFV-3 (AAA47796), and EFV (AAF64414), to build the logo.

**Figure 6:**
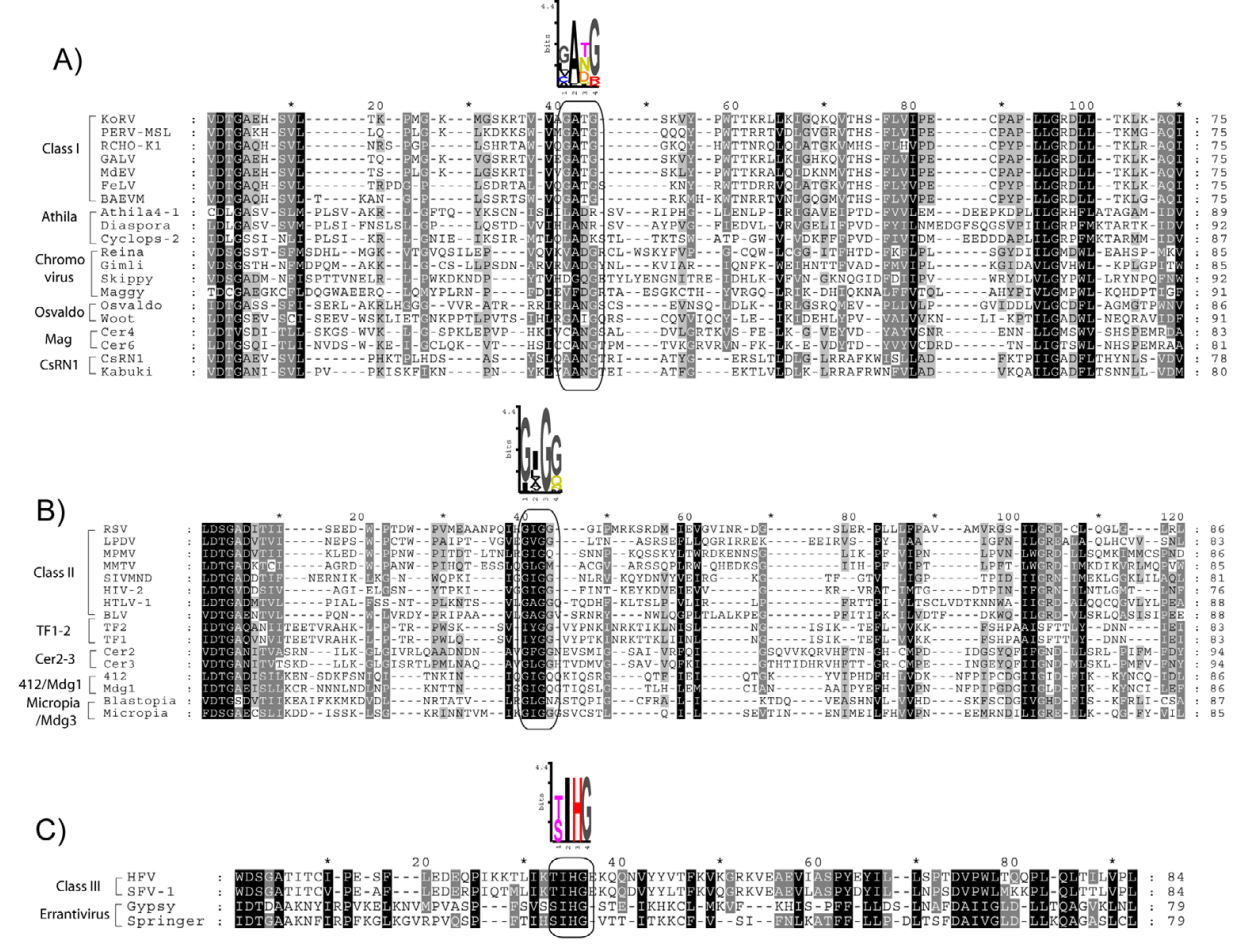
**Protease comparisons between *Ty3/Gypsy *and *Retroviridae *LTR retroelements**. Each Retroviridae PR isoform has one or more distant counterparts found among the variety of PR isoforms coded by *Ty3/Gypsy *LTR retroelements. A) Multiple alignment of class I and several *Ty3/Gypsy *lineages. This comparison reveals a similar, but not identical, flap sequence motif that in consensus defines an idealized GANG motif (logo above). B) Multiple alignment showing how the *Micropia/Mdg3 *clade and other *Ty3/Gypsy *LTR retroelements code for PRs harboring a GIGG flap variant almost identical to that of class II PRs. C) Multiple alignment between spumaretroviruses and errantiviruses showing how these 2 lineages commonly code for PRs bearing the TIHG variant.

Prior research performed using structure-based alignments and structural comparisons based on HIV-1 PR and other retropepsins, have revealed how LTR retroelement PRs dimerize in their active form (for a more extensive review in this topic, see [35] and references therein). Each lobe of the PR dimer carries a structural feature called the flap, which is a β-hairpin loop that covers the active site and has 2 flexible alternating forms, closed and semi-open (see Figure 5). We have extensively studied not only Ty3/Gypsy and Retroviridae PRs but also other clan AA PRs (data not shown). Interestingly, the *Retroviridae *differentiation into classes reveals 3 PR isoforms each preserving a particular flap motif. Class II PRs usually harbor a sequence GIGG amino acid motif (Figure 5A), which at the tertiary structure level constitute the flap in HIV-1 PR and other class II PRs (see [35] and references therein). In contrast class I PRs were found to preserve a GATG variant of this motif (Figure 5B), and within class III spumaretroviral PRs preserve a TIHG variant of the same sequence motif (Figure 5C). *Ty3/Gypsy *LTR retroelements also code for a variety of isoforms, which evolutionarily preserve a particular flap motif state depending on the lineage, in the same manner as classes I, II and III. A number of these states are very similar but not identical to that preserved by class I. Multiple alignment of gammaretroviruses (class I) and several *Ty3/Gypsy *lineages based on PR is shown in Figure 6A. In its consensus form, this variant delineates a GANG motif recognizable by the predominance of an alanine (or a hydrophobic residue) and an aspartate/asparagine/threonine at the second and third positions of the motif, respectively. The GANG variant is widespread among the PRs coded by *Ty3/Gypsy *LTR retroelements of plants, fungi and animals. This variant also predominates in the PRs coded by caulimoviruses of plants and *Ty1/Copia *LTR retroelements, and two datasets of prokaryotic PRs related to clan AA (data not shown). Therefore, GANG variant appears to be the most likely ancestral state of the flap of the PRs coded by *Ty3/Gypsy *ancestors predating the split between plants and the ophistokonts. Consistent with gag evidence, GIGG and TIHG PR variants exhibited by classes II and III PRs are rarely observed among *Ty3/Gypsy *LTR retroelements of plants and fungi. In plants, only *Tat *clade elements code for PRs presenting a poorly preserved flap motif, which might be discretely related to the GIGG variant (data not shown). As *Athila *clade elements (the sibling of *Tat *clade in plants) code for GANG PRs, we may assume that the PR flap motif transits from one state to another. Among the *Ty3/Gypsy *lineages of fungi, only TF1-2 clade code for GIGG PRs, which is a variant more frequently observed among *Ty3/Gypsy *LTR retroelements of protostomes. In contrast, the GIGG variant carried by the PRs coded by *Micropia/Mdg3 *clade and other *Ty3/Gypsy *lineages is almost identical to that of *Retroviridae *class II (Figure 6B). The TIHG variant is absent from the Ty3/Gypsy PRs of plants. In fungi, only a putative chromoviral lineage called *Ty3 *clade (see [17] and references therein) code for PRs harboring a highly diverged motif that in its consensus form can be distantly related to the TIHG variant (data not shown). In contrast, a number of *Ty3/Gypsy *errantiviruses code for PRs carrying a TIHG motif identical to that of class III spumaretroviruses (Figure 6C). Finally, investigating other sequences not considered in this study, we also found that *Gmr-1 *clade [36,37] a *Ty3/Gypsy *lineage recently described in deuterostomes also code for TIHG PRs (data not shown). The PR scenario thus reveals consistency with gag in suggesting that *Retroviridae *class I is most likely related to the phenotype of *Ty3/Gypsy *ancestors predating the spilt between plants and the ophistokonts. In contrast, classes II and IIIshould be more properly related to *Ty3/Gypsy *lineages whose ancestors probably emerged before or during the transition of bilateria organisms into protostomes and deuterostomes.

### Retroviridae class I is a molecular fossil preserving GPY/F module phenotypes that probably were predominant among *Ty3/Gypsy *ancestors predating the split between plants fungi and animals

**Figure 7:**
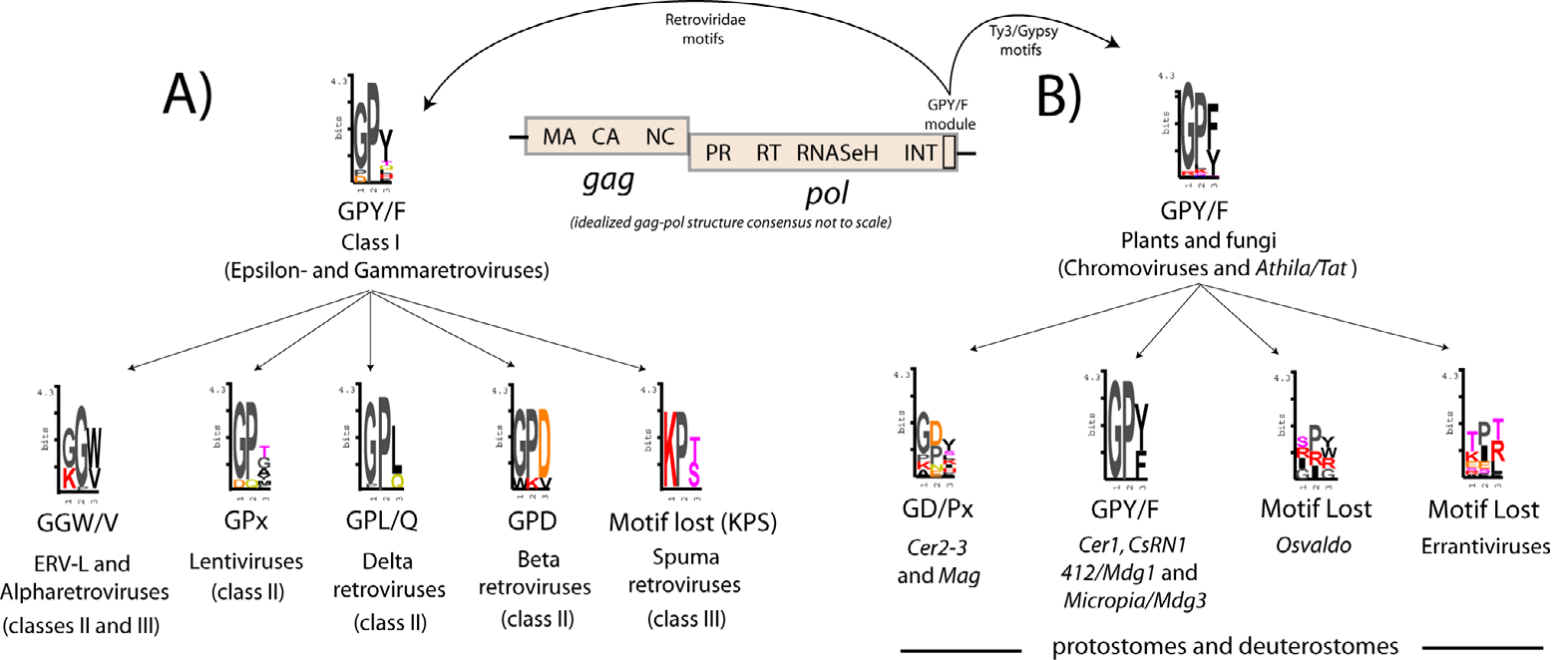
**GPY/F motif transitions**. The amino acid motif that gives its name to the GPY/F module at INT of *Ty3/Gypsy *and *Retroviridae *LTR retroelements is polymorphic. While the modules of *Retroviridae *class I and *Ty3/Gypsy *elements of plants and fungi, usually preserve the canonical GPY/F motif, classes II and III, and bilateria *Ty3/Gypsy *LTR retroelements display a number of module isoforms based on that motif.

**Figure 8:**
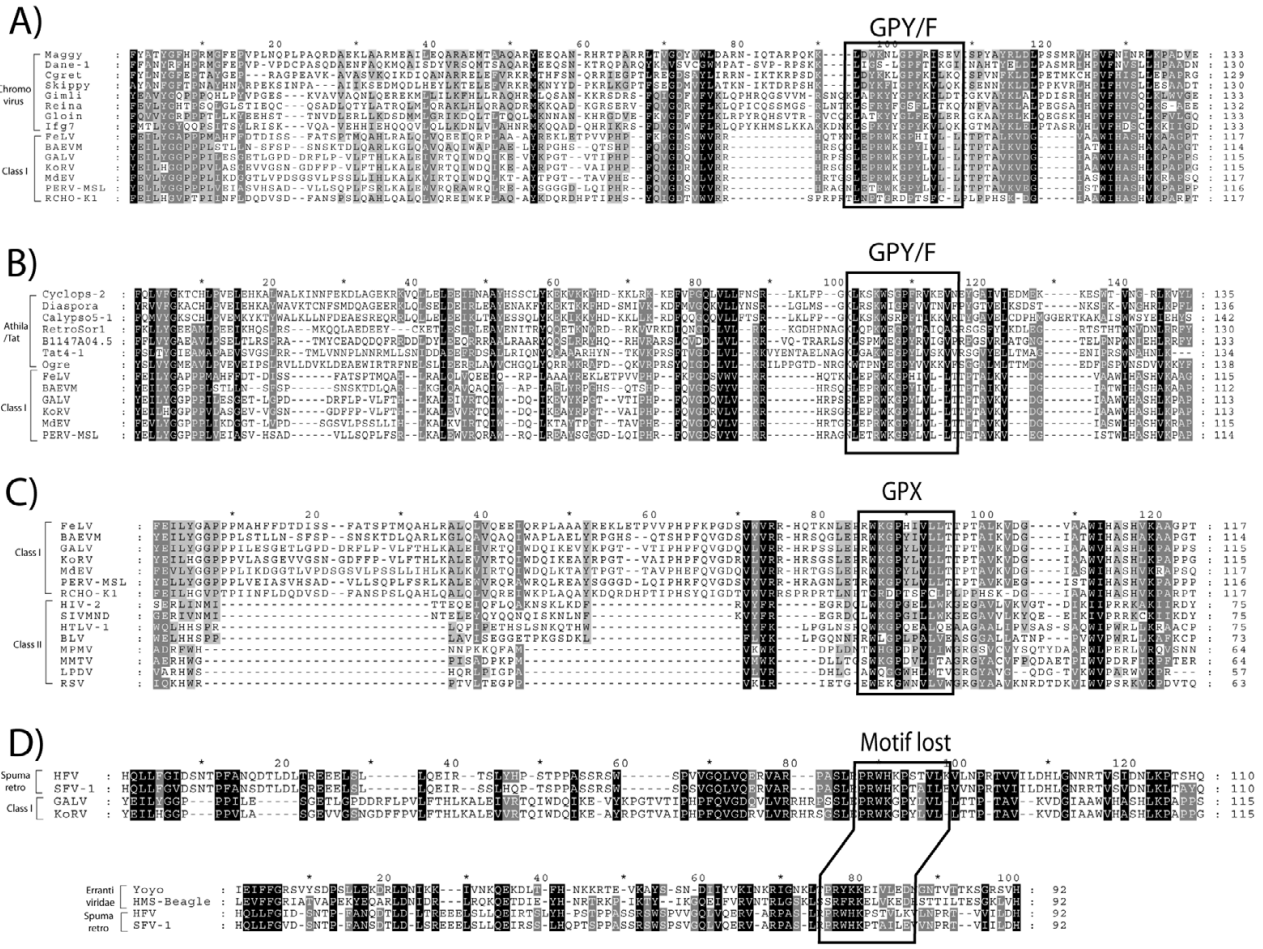
**GPY/F module comparisons**. Based on the GPY/F module, *Retroviridae *class I appears to be more similar to *Ty3/Gypsy *LTR retroelements of plants and fungi than other *Retroviridae *classes. A) Multiple alignment of gammaretroviruses (class I) and chromoviruses. B) Between gammaretroviruses and *Athila/Tat *elements. C) Between gammaretroviruses and *Retroviridae *class II, this alignment reveals important differences between the two classes, as well as the evolutionary transition of the canonical GPY/F motif towards other motif states. D) Based on the GPY/F module, spumaretroviruses (class III) is similar to class I, but also to *Ty3/Gypsy *errantiviruses. In fact, both spumaretroviral and errantiviral modules lost the canonical motif during evolution.

As already shown, gag polyprotein and the PR depict a new scenario as an alternative to the traditional monophyletic insight (2 groups of LTR retroelements) suggested by prior RT, RNAse H and INT analyses. Onto understand the two opposing scenarios, we performed phylogenetic analyses based on the RT, RNAse H and INT and found consistency with the traditional perspective of 2 separate LTR retroelement groups using the RT and RNAse H ([13-15]). Analysis of the INT revealed different perspectives depending on the NJ or parsimony method used in the analysis (see Methods). While the NJ method supports the 2 LTR retroelement groups, the parsimony method splits the *Retroviridae *into 2 branches not supported by bootstrap (data not shown). This is because our model of INT alignment covers the 3 subdomains described in the amino acidic architecture of a conventional INT domain. The traditional core used for inferring INT phylogenies is common to all INTs in general, and includes 2 of these sub-domains; the conserved zinc finger "HHCC" binding motif [38] at the N-terminus, and the central sub-domain containing the conserved D-D-E trait [39,40]. The C-terminal sub-domain of all INTs is usually dismissed from analysis because it is less preserved than the other 2 sub-domains. In Ty3/Gypsy and RetroviridaeINTs, it is definite that this sub-domain is a small trait called GPY/F module, which was probably recruited modularly during evolution [14]. The module name refers to the strongly preserved GPY/F amino acid motif [14], which will be referred to as the canonical motif throughout the rest of this paper. Indeed, the GPY/F module appears to be responsible of the signal discrepancy in phylogenetic analyses (INT parsimony tree performed without this module is in agreement with the NJ analysis, data not shown). From that point, we investigated the GPY/F module in relation to the 3 *Retroviridae *classes. The module, seen from this viewpoint, shows a number of protein isoforms based on GPY/F motif polymorphisms. With rare exceptions, the modules of class I INTs usually preserve the canonical motif, while the modules of classes II and III exhibit other variants (Figure 7A). Here, classes II and III do not make an intrinsic phenotypic distinction, each genus exhibiting a particular variant of the motif within these 2 classes. The modules coded by *Ty3/Gypsy *LTR retroelements delineate similar perspective. As shown in Figure 7B, while the canonical motif is practically predominant in the modules of *Ty3/Gypsy *elements of plants and fungi, the modules of bilateria *Ty3/Gypsy *LTR retroelements are rich in motif polymorphisms (canonical motif included). This indicates that *Ty3/Gypsy *LTR retroelements described in bilateria organisms exhibit greater GPY/F motif variability than their *Ty3/Gypsy *counterparts of plants and fungi, and strongly suggests a number of transitions from the canonical motif toward other states during evolution. This scenario is not completely consistent with gag and PR perspectives; for instance, while Micropia/Mdg3 modules preserve the canonical motif, the different *Retroviridae *genera belonging to class II exhibit different motif polymorphisms. Nevertheless, the GPY/F module relates the *Retroviridae *class I with *Ty3/Gypsy *LTR retroelements of plants and fungi through the common preservation of the canonical motif, while classes II and III can be related with bilateria *Ty3/Gypsy *LTR retroelements by an increase of the motif variability. In fact, the whole module of class I INTs appears to be more similar to those preserved by the INTs of chromoviruses (Figure 8A) and *Athila *and *Tat *clades (Figure 8B) than to those of classes II and III. Alignment between classes I and II reveals a dramatic loss of sequence information by class II during evolution (Figure 8C). The module carried by spumaretroviral INTs is similar to that of class I, but they greatly differ in the motif (Figure 8D). That is, spumaretroviral modules lost the GPY/F motif, substituting it with a highly diverged KT/SP motif. Again, this outlines an intriguing parallelism between spumaretroviruses and *Ty3/Gypsy *errantiviruses because the modules of these 2 LTR retroelement lineages are qualitatively similar (Figure 8D). Moreover, *Ty3/Gypsy *errantiviruses also lost their GPY/F motif during evolution. Therefore, whatever the INT function involving the GPY/F module coded by the *Retroviridae *class I, this class appears to be a molecular fossil preserving GPY/F module phenotypes that were predominant among *Ty3/Gypsy *ancestors, predating the split between plants fungi and animals. In contrast, *Retroviridae *classes II and III maintain a number of module isoforms more recently emerged during evolution.

## Discussion

### Retroviridae differentiation into the 3 classes I, II and III unravels phenotypic aspects of vertebrate retroviruses, which are probably related with their ancient *Ty3/Gypsy *origins

Phylogenetic analysis inferred based on all concatenated gag and pol products coded by *Ty3/Gypsy *and *Retroviridae *LTR retroelements shows the robustness of their phylogenetic signal regarding the clustering of OTUs [5-14,19-25]. We used the parsimony method to infer this phylogeny, but the clustering of OTUs is independent of the method of phylogenetic reconstruction used (see Methods). The gag-pol analysis also divides *Ty3/Gypsy *and *Retroviridae *LTR retroelements into 2 separate branches, as suggested by original approaches in this topic [12,41]. We do not disagree this classification for 2 reasons; first, the strong phylogenetic signal of RT, RNAseH, and INT cannot be dismissed;and second, the *Retroviridae *(except gammaretroviruses) can be distinguished from *Ty3/Gypsy *LTR retroelements by features such as the presence of accessory genes. Nevertheless, thecurrent *Ty3/Gypsy *and *Retroviridae *classification only exposes the modern evolutionary history of these 2 groups of retroelements (we have shown how their ancient history is not straightforward). Due to the wide distribution of *Ty3/Gypsy *elements in eukaryotes, the usual means of transference of a canonical *Ty3/Gypsy *LTR retrotransposon is probably vertical. However, the viral nature of a true *Ty3/Gypsy *or *Retroviridae *exogenous retrovirus resides in its capability of horizontal transference from one host to another via infection. Moreover, the incidence of mechanisms such as gene recruitment, genome rearrangement, recombination and chimerism in LTR retroelement evolution, presents difficulties in identifying the true natural history of *Ty3/Gypsy *and *Retroviridae *LTR retroelements. This suggests that the most realistic (not yet proposed) model for describing *Ty3/Gypsy *and *Retroviridae *evolution alternates gradual and modular evolution, and combines vertical and horizontal means of transference.

The traditional argument supporting the *Ty3/Gypsy *origins of vertebrate retroviruses is shown by their similarity in sequence and genome structure [41]. The question is, however, what genetic material is more informative for exploring the relationships between these two (and other) groupsof LTR retroelements, highl y variable traits such as gag and PR or strongly preserved substrates such as the RT, RNAseH and INT? Certainly, RT, RNAseH and INT are an excellent means of classifying *Ty3/Gypsy *and *Retroviridae *LTR retroelements into lineages. However, phylogenetic analyses based on RT, RNAseH and INT are not exact enough to resolve the ancient evolutionary history of these 2 groups. This is because the inferred phylogeny based on these proteins does not necessarily coincide with the true natural history of the full-length retroelement genome. Here, the advantage of using the gag-pol alignment to infer the phylogeny is the increase in statistical power of the analysis, allowing the opportunity to correct the single gene tree discrepancies. This analytical strategy is useful but has limitations for which solutions remain elusive; the inferred tree can accumulate systematic errors due to the use of concatenated information. We have shown how gag-pol tree suggests a *Ty3/Gypsy *root in the origins of vertebrate retroviruses that is close to the *Micropia/Mdg3 *clade. However, evaluation of gag and pol polyproteins separately yields discordant information. Here, while pol phylogeny supports the traditional perspective (2 retroelement groups), gag phylogeny describes a new scenario that appears to be informative with respect to the ancient patterns of diversity of *Ty3/Gypsy *and *Retroviridae *LTR retroelements. Certainly, the phylogenetic signal of the gag polyprotein has several limitations due to its fast evolution. To overcome these limitations we investigated other protein domains and used different methodologies to evaluate the significance of the new scenario. The most important feature here is that, for first time in the scientific literature, we have carried out a non-redundant study of three independent proteins that have rarely been attempted before because their difficulty.

Our investigation conclusively reveals that the taxonomical differentiation into the 3 *Retroviridae *classes I, II and III discloses 3 different gag and PR products, and that each product has one or more distant *Ty3/Gypsy *counterparts. The analysis of the GPY/F module reveals partial consistency and how the similarity of class I to *Ty3/Gypsy *LTR retroelements of plant and fungi, is significant. Our results thus support an ancient scenario of polyphyly involving the 3 *Retroviridae *classes and different *Ty3/Gypsy *lineages. Here, we stress that the identification of the *Retroviridae *classes is not a conclusion but an assumption based on previous studies [6-10]. Notwithstanding, we cannot argue for the existence of a direct ancestor between each class and any particular *Ty3/Gypsy *lineage. Classes I and II are sufficiently similar to corroborate their accepted evolutionary relationship, and it can also be assumed that *Ty3/Gypsy *and *Retroviridae *phylogeny is incomplete (sequencing projects are continuously disclosing new lineages). Despite this, the similarity of each class by simple convergence to different *Ty3/Gypsy *lineages based on 3 independent protein products is an implausible parsimonious explanation. Moreover, while class III spumaretroviruses are dissimilar to classes I and II, our results reveal that they in turn display an intriguing domain similarity to errantiviruses that ought to be followed up. Hence we think that the class differentiation probably unravels certain aspects of vertebrate retroviruses related to their ancient *Ty3/Gypsy *origins. Instead of a single root to this new scenario, we show how an ancient evolutionary network between the 2 groups can exist, with its most interesting aspect being its polyphyly. (The *Ty3/Gypsy *lineages related to each class does not constitute a monophyletic branch in any phylogeny). Therefore, our approach strongly suggest that class I is a molecular fossil that emerged quite soon in *Ty3/Gypsy *evolution, while classes II and III emerged later, together with the ancestors of *Ty3/Gypsy *LTR retroelements described in protostomes.

### Introducing the Three Kings Hypothesis: A new principle for debate and further evaluation about the subject of the *Ty3/Gypsy *origins of vertebrate retroviruses

The evolutionary network identified by classes I, II, III is inconsistent with the idea of a unique *Retroviridae *ancestor. It follows that various scenarios may either support or disprove such a network. Assuming this network exists, the most likely scenario relates *Ty3/Gypsy *elements of plants and fungi with the *Retroviridae *class I. This scenario assumes the existence of a distant evolutionary relationship between the lineages or an ancient horizontal transfer of chromoviruses from fungi (or plants) to vertebrates. Indeed, chromoviruses are the most ancient lineage of *Ty3/Gypsy *LTR retrotransposons. They are rich in genetic variability, and are also present in the genome of many vertebrates [23,32,33]. In both cases, the most likely explanation for the relationship between class I and *Athila/Tat *retroviruses and retrotransposons of plants is that chromoviruses and class I are related, an argument suggested by a previous study [30]. Nevertheless, chromoviruses of vertebrate organisms are usually more similar to their chromoviral counterparts of fungi than to those of plants. Therefore the chromoviral scenario does not explain why class I and *Athila/Tat *elements of plants are similar each other based on gag. On the other hand, chromoviruses have not yet been described in protostomes, echinoderms and urochordates; furthermore it remains unclear whether chromoviruses were inexorably driven to extinction in these organisms or were horizontally transmitted from plants/fungi to vertebrates. Consequently, the chromoviral scenario does not clarify why classes II and III and the *Ty3/Gypsy *lineages of protostomes share sequence similarities and phenotypic features rarely found among the *Ty3/Gypsy *lineages of plants and fungi. With this in mind, a new theoretical principle is posited here for debate and further research. The simplest hypothesis is that classes I, II and III probably evolved from at least 3 *Ty3/Gypsy *ancestors and emerged at different evolutionary times prior to the split between protostomes and deuterostomes (*the three kings hypothesis*). Several points involved in the background of this hypothesis should be emphasized. First, we include the words "at least" to acknowledge the three classes but do not dismiss the possibility of more *Ty3/Gypsy *ancestors in the evolutionary history of the *Retroviridae*. Second, "different times of emergence" suggests, but does not necessarily mean, independent origins. Class II may in fact be directly related to class I, but the emergence of class II seems more recent and in parallel with the emergence of the ancestorsof several *Ty3/Gypsy *lineages, such as the *Micropia/Mdg3 *clade (or others). Class III spumaretroviruses delineate identical perspective with *Ty3/Gypsy *errantiviruses. Third, we use the term "polyphyletic" because the *Ty3/Gypsy *lineages related to each class do not constitute a monophyletic branch in any phylogeny. Moreover, viral evolution is always a polyphyletic challenge involving ecological parameters such as host populations, environment, vectors, mechanisms of transmissions, etc.

### The polyphyletic recurrence of vertebrate retroviruses into the evolutionary performance of *Ty3/Gypsy *LTR retroelements

We have described how the different gags, PRs and GPY/F modules evaluated show a variability that is preserved, depending on the *Ty3/Gypsy *lineage and *Retroviridae *class (or genus). While class I can be related to *Ty3/Gypsy *elements of plants and fungi, classes II and III preserve phenotypic features typically observed among *Ty3/Gypsy *elements of protostomes. That is the evolutionary perspective provided by the protein product of 3 independent coding regions. We have discussed this evidence but have not yet interpreted why the diversity and phylogeny of *Ty3/Gypsy *and *Retroviridae *LTR retroelements are so different regarding the different gag or pol substrates. In general, the action of viruses and mobile genetic elements is important in host evolution [16,42-47] because they are vectors of evolution and potential inducers of diseases and genetic disorders, such as chromosome rearrangements and inversions [48]. However, if the action of viruses and mobile genetic elements might somehow influence the host evolution, it is reasonable that host evolution could also constrain the evolution of these genetic agents. We thus speculate with the possibility of selective influences imposed on *Retroviridae *genes such as the *rt, rnase h *and *int *(and other regions) to optimize essential functions, such as retrotranscription and integration (according to the complexity of the new genome environment provided by vertebrate organisms). This probably involves gradual evolution but also a number of molecular mechanisms, such as gene recruitment and recombination to generate variability and new effective genetic combinations. Here, it is important to keep in mind that except gammaretroviruses and other exceptions, the *Retroviridae *usually incorporate accessory genes, usually needed to adjust diverse aspects of their replication and infectivity (these features appear to be specific of retroviruses infecting vertebrate organisms). On the other hand, a prior study [15] supports a putative chimeric origin of the *Retroviridae *RNAse H domain and the modular acquisition of the GPY/F module by Ty3/Gypsy and Retroviridae INTs [14]. Moreover, D-type betaretroviruses probably are viral hybrids between a B-type betaretrovirus and a C-type gammaretrovirus [5,17,49]. Finally, a number of studies reveal how recombination is a mechanism frequently embraced by HIV evolution to generate variability. Two studies reveal for instance how recombination of M subtypes, has resulted in the generation of multiple circulating recombinant forms consisting of mosaic HIV-1 lineages [50,51].

Regarding coding regions such as *gag, pr *and *gpy/f *module, we think that these traits reveal features and aspects involving different evolutionary strategies, but which are intrinsic and taxonomically related with ancient events of retroelement speciation and divergence. This argument finds an important evolutionary marker in the variability in the number of CCHC arrays at NC and the different PR and GPY/F module isoforms. Indeed, the CCHC array at NC is involved in virion assembly, RNA packaging, reverse transcription and integration processes [52]. On the other hand, the flap lies over the PR active site and conveys specificity to the enzyme by carrying important substrate-binding functions (for more information in this topic, see [35,53,54]). Finally, while the GPY/F module is now under investigation, the C-terminal end of the INT appears to be important in the integration of the retroelement into the host genome [55,56]. The variability of these three regions probably reveals different evolutionary strategies of speciation and divergence, which can be assumed older than previously supposed, since it does not only occur in the *Retroviridae *group, but also in all *Ty3/Gypsy *LTR retroelements of plants, fungi and animals. Here, the *three kings hypothesis *and its testing (in one sense or another) does not affect the evidence we have presented. That is, class I, II and III taxonomically code for 3 gag, PR and GPY/F products that have one or more distant counterparts among *Ty3/Gypsy *LTR retroelements. However, the most interesting aspect of the gag-PR-GPY/F variability is that it appears to be constrained by the bio-distribution of *Ty3/Gypsy *LTR retroelements. In turn, the diversity patterns of the *Retroviridae *based on these regions appear to be recurrent into the evolutionary performance of *Ty3/Gypsy *LTR retroelements, the most interesting aspect of which is that they seem polyphyletic. Therefore the evolutionary network between *Ty3/Gypsy *and *Retroviridae *LTR retroelements is informative regarding an ancestral history, which is in some respects similar to those models of evolution indistinctly described by population genetics and quasi-species theory (for more details see [57]). This means that further analysis of the evolutionary network we disclose in this study challenges the involvement of different parameters such as bio-distribution, host's populations, environment, vectors and mechanisms of transmissions, etc. With this aim, our hypothesis makes possible a first evaluation of this new scenario we present in a forthcoming manuscript (submitted for publication). In this approach, we use the number of CCHC arrays at NC and the different PR and GPY/F module isoforms as evolutionary markers to trace the network. This is by superimposing not only *Ty3/Gypsy *and *Retroviridae *LTR retroelements, but also other LTR retroelement groups over their host bio-distribution.

## Conclusion

*Retroviridae *classes I, II and III exhibit phenotypic differences that delineate a network never before reported between *Ty3/Gypsy *and *Retroviridae *LTR retroelements. This new scenario reveals how the diversity of vertebrate retroviruses is polyphyletically recurrent into the *Ty3/Gypsy *evolution, i.e. older than previously thought. The simplest hypothesis to explain this finding is that classes I, II and III trace back to at least 3 *Ty3/Gypsy *ancestors that emerged at different evolutionary times prior to protostomes-deuterostomes divergence. We have called this "the three kings hypothesis" concerning the origin of vertebrate retroviruses.

## Methods

### Sequences and databases

This work is part of the GyDB Project [17] an ongoing database launched with the aim of phylogenetically analyzing and classifying mobile genetic elements based on their diversity and evolutionary profile. In the first iteration, we consider the *Ty3/Gypsy *and *Retroviridae *LTR retroelements of eukaryotes. We have investigated 120 non-redundant full-length *Ty3/Gypsy *and *Retroviridae *genomes collected from NCBI [58]. An extended version of the gag-pol tree evaluated summarizing names, taxonomy, hosts, and Genbank accessions of all retroelement taxa used to perform this analysis, is available online as the Additional file 1 accompanying this paper. By clicking the name of each OTU in this tree, the user can browse the GyDB and locate a file providing information of the OTU selected, including a link to the Genbank accession of the requested element at NCBI. The gag-pol tree can also be found online in the Section Phylogenies at GyDB [59].

### Multiple alignments and comparative analyses

In general, all *Ty3/Gypsy *and *Retroviridae *LTR retroelements have 2 polyproteins in common – gag and pol. Gag is composed of 3 domains -MA, CA and NC -, pol is usually carrier of 4 domains – PR, RT, RNAse H and INT. Note however that PR can be coded separately or in frame with gag and other protein domains. We have used and analyzed a gag-pol multiple alignment ~1700 residues in size, constructed based on the concatenation of the CA, NC, PR, RT, RNAseH and INT cores. The gag-pol alignment is freely accessible within the GyDB collection deposited at Biotechvana Bioinformatics [60]. The alignment is available in 6 formats at the following URL [61]. We have also analyzed the gag and pol polyproteins by separate dividing the gag-pol alignment into 2 independent alignments CA-NC and PR-RT-RNAseH-INT, to perform phylogenetic or comparative analyses.

Alignments were compared using GENEDOC editor [62] in shaded mode and the following groups of amino acid similarity: [T,S small nucleophile amino acids] [K,R,H basic amino acids], [D,E,N,Q acidic amino acid and relative amides], and [L,I,V,M,A,G,P,F,Y,W hydrophobic amino acids]. Similarities between gag sequences were correlated using different gag queries to the CORES database available via the NCBI BLAST search [29] at GyDB, using BLASTp search mode. BLAST databases available at GyDB are non-redundant, small and include only *Ty3/Gypsy *and *Retroviridae *or related sequences, allowing flexible comparisons between both distantly and closely related sequences with homologous known functions.

Comparative analyses based on sequence logos involved CheckAlign 1.0 [63] in Shannon's algorithm mode [64] and correction factor. Sequence logomethodology was originally introduced by Schneider et al. [65,66] to display consensus sequences for DNA and protein alignments. Later, Schneider dismissed the term "consensus" [67], arguing that a logo provides more information than the consensus sequence of a protein or DNA alignment. While this can be controversial because there are many manners to obtain or describe a consensus sequence, logos methodology being one of them, we are in agreement with the proposition of the original author in the use of the term "sequence logo" suggested in his website [68]. We employ the term "sequence logo" to describe the resultant output reported by this analysis, and then refer to the protein information underlying the content shape of the logos constructed, based on our alignments as "amino acidic architecture". This term may be useful to describe with a single word – consensus, core and amino acid patterns. CheckAlign directly builds the logo from an ungapped alignment using the conventional methodology [65,66]. Here, the maximum uncertainty by position in a protein alignment is log_2 _20 = 4.3. In the case of gapped alignments, CheckAlign automatically builds the logo, taking the gap as another amino acid species. Here, the tool considers the maximum uncertainty by position to be log_2 _21 = 4.4 for protein alignments (for more details about CheckAlign see [63]).

The 3D structure of the HIV-1 PR [69] was modeled using SWISS-PDBViewer 3.7 SP5 [70], and PDB file 1A30 as input. The PDB file was downloaded from RCSB Protein Data Bank [71].

### Phylogenetic analyses

Phylogenetic reconstructions of *Ty3/Gypsy *and *Retroviridae *LTR retroelements inferred from gag-pol, pol and gag alignments employed the PHYLIP 3.6 package [72]. We first generated 100 bootstrap replicates of each alignment using SEQBOOT. Second, we used the protein sequence parsimony method of Felsenstein, based on the approaches of Eck and Dayhoff [73] and Fitch [74] to perform the analyses. Here, the bootstrap file was used as an input to PROTPARS and the input randomized using the following parameters, random number seed = 5 and number of times to jumble = 5. Third, CONSENSE was used to obtain a MRC tree [75] using the tree file generated by PROTPARS as an input. As the MRC tree usually consists of all clusters that occur >50% of the time, we took consensus values >55 as a bootstrap reference. Bootstrap values were used to scale the trees.

We also tested the NJ method [76] using different models of distances implemented in PROTDIST. Here, it is important to keep in mind that the overall efficiency of the different methods of phylogenetic reconstruction in building the true tree vary with substitution rate, transition-transversion ratio, and sequence divergence [77,78]. With the particular material we studied, parsimony and NJ trees support the clustering of OTUs into clades and genera in gag-pol and pol analyses, and they are consistent in not supporting the monophyly of each group in gag analyses. However, parsimony phylogenies proved more consistent with comparative analyses than NJ trees when inferring phylogenies including or evaluating the gag and/or PR proteins. Parsimony analyses also reported better bootstrapping and were more consistent with the three *Retroviridae *classes than NJ analyses (NJ trees only support classes I and II).

## Abbreviations

(AIDS): Acquired Immune Deficiency Syndrome; (BLV): Bovine Leukemia Virus; (CA): Capsid; (CAEV): Caprine Arthritis Encephalitis Virus; (EFV): Equine Foamy Virus; (FeLV): Feline Leukemia Virus; (FFV): Feline Foamy Virus; (FSV): Feline Syncytial Virus; (ICTV): International Committee on Taxonomy of Viruses; (GyDB): Gypsy Database; (HIV): Human Immunodeficiency Virus; (HFV): Human Foamy Virus; (HTLV): Human T-cell Leukemia Virus; (HMM profile): Hidden Markov Model; (INT): Integrase; (LTR): Long terminal repeat; (LPDV): Lymphoproliferative Disease Virus; (MHR): Major homology region; (VMV): Maedi Visna Virus; (MRC): Majority-rule consensus; (MPMV): Mason-Pfizer Monkey Virus; (MA): Matrix; (MMTV): Mouse Mammary Tumor Virus; (NCBI): National Center of Biotechnology Information; (NJ): Neighbor joining; (NC): Nucleocapsid; (OTU): Operative taxonomical unit ; (SA-OMVV): Ovine Maedi Visna Virus; (PR): Protease; (RCSB): Research Collaboratory for Structural Bioinformatics; (RT): Reverse transcriptase; (RNAse H): Ribonuclease H; (RSV): Rous Sarcoma Virus; (SCSIE): Servei Central de Suport a la Investigació Experimental; (SERV): Simian Endogenous Retrovirus of Mandrill; (SIVMAC): Simian Immunodeficiency Retrovirus of Macaques; Simian Foamy Virus (SFV); (SRV): Simian Retrovirus; (3D structure): Three-dimensional structure.

## Authors' contributions

CL and AM conceived and designed the study. CL performed the analyses and CL and MAF wrote the paper.

## Supplementary Material

Additional File 1 **Expanding gag-pol phylogeny.** Expanded version of gag-pol tree illustrated in Figure 1 inferred based on the 120 *Ty3/Gypsy *and *Retroviridae *LTR retroelements used in this study. The tree includes information about the names, Genbank accessions and hosts of all LTR retroelement taxa used. By clicking the name of each OTU, the user can locate a file at GyDB providing information of the sequence selected, including a link to its Genbank accession at NCBI. Click here for file

## References

[B1] Poiesz BJ Ruscetti FW Gazdar AF Bunn PA Minna JD Gallo RC Detection and isolation of type C retrovirus particles from fresh and cultured lymphocytes of a patient with cutaneous T-cell lymphoma Proc Natl Acad Sci USA 1980 77 7415 7419 10.1073/pnas.77.12.7415 6261256 PMC350514

[B2] Yoshida M Miyoshi I Hinuma Y Isolation and characterization of retrovirus from cell lines of human adult T-cell leukemia and its implication in the disease Proc Natl Acad Sci USA 1982 79 2031 2035 10.1073/pnas.79.6.2031 6979048 PMC346116

[B3] Barre-Sinoussi F Chermann JC Rey F Nugeyre MT Chamaret S Gruest J Dauguet C xler-Blin C Vezinet-Brun F Rouzioux C Isolation of a T-lymphotropic retrovirus from a patient at risk for acquired immune deficiency syndrome (AIDS) Science 1983 220 868 871 10.1126/science.6189183 6189183

[B4] Gallo RC Salahuddin SZ Popovic M Shearer GM Kaplan M Haynes BF Palker TJ Redfield R Oleske J Safai B Frequent detection and isolation of cytopathic retroviruses (HTLV-III) from patients with AIDS and at risk for AIDS Science 1984 224 500 503 10.1126/science.6200936 6200936

[B5] Van Regenmortel MHV Fauquet CM Bishop DHL Carstens EB Estes MK Lemon SM Maniloff J Mayo MA McGeoch DJ Pringle CR Wickner RB Virus Taxonomy: the classification and nomenclature of viruses 2000 San Diego, California

[B6] International Human Genome Consortium Initial sequencing and analysis of the human genome Nature 2001 409 860 921 10.1038/35057062 11237011

[B7] International Human Genome Consortium Initial sequencing and analysis of the human genome Nature 2002 420 520 562 10.1038/nature01262 12466850

[B8] Wilkinson DA Mager DL Leong JA Levy JA Endogenous Human Retroviruses The Retroviridae 1994 II New York, N.Y.: Plenum Press, Inc 465 535

[B9] Gifford R Tristem M The evolution, distribution and diversity of endogenous retroviruses Virus Genes 2003 26 291 315 10.1023/A:1024455415443 12876457

[B10] Gifford R Kabat P Martin J Lynch C Tristem M Evolution and distribution of class II-related endogenous retroviruses J Virol 2005 79 6478 6486 10.1128/JVI.79.10.6478-6486.2005 15858031 PMC1091674

[B11] Eickbush TH Malik HS Craig NL, Craigie R, Gellert M, Lambowitz AM Origin and Evolution of retrotransposons Mobile DNA II 2002 Washington DC.: ASM Press 1111 1144

[B12] Xiong Y Eickbush TH Origin and evolution of retroelements based upon their reverse transcriptase sequences EMBO J 1990 9 3353 3362 1698615 10.1002/j.1460-2075.1990.tb07536.x PMC552073

[B13] Marin I Llorens C Ty3/Gypsy retrotransposons: description of new Arabidopsis thaliana elements and evolutionary perspectives derived from comparative genomic data Mol Biol Evol 2000 17 1040 1049 10889217 10.1093/oxfordjournals.molbev.a026385

[B14] Malik HS Eickbush TH Modular evolution of the integrase domain in the Ty3/Gypsy class of LTR retrotransposons J Virol 1999 73 5186 5190 10233986 10.1128/jvi.73.6.5186-5190.1999 PMC112568

[B15] Malik HS Eickbush TH Phylogenetic analysis of ribonuclease H domains suggests a late, chimeric origin of LTR retrotransposable elements and retroviruses Genome Res 2001 11 1187 1197 10.1101/gr.185101 11435400

[B16] Llorens C Marin I A mammalian gene evolved from the integrase domain of an LTR retrotransposon Mol Biol Evol 2001 18 1597 1600 11470852 10.1093/oxfordjournals.molbev.a003947

[B17] Llorens C Futami R Bezemer D Moya A The Gypsy Database (GyDB) of Mobile Genetic Elements Nucleic Acids Research (NAR) 2008 36 38 46 10.1093/nar/gkm697 PMC2238898 17895280

[B18] Eddy SR Profile hidden Markov models Bioinformatics 1998 14 755 763 10.1093/bioinformatics/14.9.755 9918945

[B19] Wright DA Voytas DF Potential retroviruses in plants: Tat1 is related to a group of Arabidopsis thaliana Ty3/gypsy retrotransposons that encode envelope-like proteins Genetics 1998 149 703 715 9611185 10.1093/genetics/149.2.703 PMC1460185

[B20] Wright DA Voytas DF Athila4 of Arabidopsis and Calypso of soybean define a lineage of endogenous plant retroviruses Genome Res 2002 12 122 131 10.1101/gr.196001 11779837 PMC155253

[B21] Bae YA Moon SY Kong Y Cho SY Rhyu MG CsRn1, a novel active retrotransposon in a parasitic trematode, Clonorchis sinensis, discloses a new phylogenetic clade of Ty3/gypsy-like LTR retrotransposons Mol Biol Evol 2001 18 1474 1483 11470838 10.1093/oxfordjournals.molbev.a003933

[B22] Bowen NJ McDonald JF Genomic analysis of Caenorhabditis elegans reveals ancient families of retroviral-like elements Genome Research 1999 9 924 935 10.1101/gr.9.10.924 10523521

[B23] Gorinsek B Gubensek F Kordis D Evolutionary genomics of chromoviruses in eukaryotes Mol Biol Evol 2004 21 781 798 10.1093/molbev/msh057 14739248

[B24] Britten RJ Active gypsy/Ty3 retrotransposons or retroviruses in Caenorhabditis elegans Proc Natl Acad Sci USA 1995 92 599 601 10.1073/pnas.92.2.599 7530364 PMC42789

[B25] Ganko EW Fielman KT MacDonald JF Evolutionary History of Cer Elements and Their Impact on the C.elegans genome Genome Res 2001 11 2066 2074 10.1101/gr.196201 11731497 PMC311226

[B26] Pringle CR Virus taxonomy, The Universal System of Virus Taxonomy, updated to include the new proposals ratified by the International Committee on Taxonomy of Viruses during 1998 Archives of Virology 1999 144 421 429 10.1007/s007050050515 10470265 PMC7086988

[B27] Boeke JD Eickbush TH Sandmeyer SB Voytas DF Metaviridae Virus Taxonomy: ICTV VIIth report 1999 Springer-Verlag, New York

[B28] Hull R Classification of reverse transcribing elements: a discussion document Archives of Virology 1999 144 209 214 10.1007/s007050050498 10076522

[B29] Altschul SF Madden TL Schaffer AA Zhang J Zhang Z Miller W Lipman DJ Gapped BLAST and PSI-BLAST: a new generation of protein database search programs Nucleic Acids Res 1997 25 3389 3402 10.1093/nar/25.17.3389 9254694 PMC146917

[B30] Nakayashiki H Matsuo H Chuma I Ikeda K Betsuyaku S Kusaba M Tosa Y Mayama S Pyret, a Ty3/Gypsy retrotransposon in Magnaporthe grisea contains an extra domain between the nucleocapsid and protease domains Nucleic Acids Res 2001 29 4106 4113 10.1093/nar/29.20.4106 11600699 PMC60222

[B31] Green LM Berg JM A retroviral Cys-Xaa2-Cys-Xaa4-His-Xaa4-Cys peptide binds metal ions: spectroscopic studies and a proposed three-dimensional structure Proc Natl Acad Sci USA 1989 86 4047 4051 10.1073/pnas.86.11.4047 2786206 PMC287385

[B32] Gorinsek B Gubensek F Kordis D Phylogenomic analysis of chromoviruses Cytogenet Genome Res 2005 110 543 552 10.1159/000084987 16093707

[B33] Kordis D A genomic perspective on the chromodomain-containing retrotransposons: Chromoviruses Gene 2005 347 161 173 10.1016/j.gene.2004.12.017 15777633

[B34] Rawlings ND Tolle DP Barrett AJ MEROPS: the peptidase database Nucleic Acids Research 2004 32 D160 D164 10.1093/nar/gkh071 14681384 PMC308805

[B35] Wlodawer A Gustchina A Structural and biochemical studies of retroviral proteases Biochim Biophys Acta 2000 1477 16 34 10708846 10.1016/s0167-4838(99)00267-8

[B36] Butler M Goodwin T Poulter R An unusual vertebrate LTR retrotransposon from the cod Gadus morhua Mol Biol Evol 2001 18 443 447 11230547 10.1093/oxfordjournals.molbev.a003822

[B37] Goodwin TJ Poulter RT A group of deuterostome Ty3/gypsy-like retrotransposons with Ty1/copia-like pol-domain orders Mol Genet Genomics 2002 267 481 491 10.1007/s00438-002-0679-0 12111555

[B38] Lodi PJ Ernst JA Kuszewski J Hickman AB Engelman A Craigie R Clore GM Gronenborn AM Solution structure of the DNA binding domain of HIV-1 integrase Biochemistry 1995 34 9826 9833 10.1021/bi00031a002 7632683

[B39] Polard P Chandler M Bacterial transposases and retroviral integrases Mol Microbiol 1995 15 13 23 10.1111/j.1365-2958.1995.tb02217.x 7752887

[B40] Khan E Mack JP Katz RA Kulkosky J Skalka AM Retroviral integrase domains: DNA binding and the recognition of LTR sequences Nucleic Acids Res 1991 19 851 860 10.1093/nar/19.4.851 1850126 PMC333721

[B41] Eickbush TH Morse SS Origin and evolutionary relationships of LTR retroelements The evolutionary Biology of viruses 1994 New York: Raven 121 157

[B42] Lynch M Conery JS The origins of genome complexity Science 2003 302 1401 1404 10.1126/science.1089370 14631042

[B43] Ganko EW Bhattacharjee V Schliekelman P McDonald JF Evidence for the contribution of LTR retrotransposons to C. elegans gene evolution Mol Biol Evol 2003 20 1925 1931 10.1093/molbev/msg200 12885961

[B44] Brandt J Schrauth S Veith AM Froschauer A Haneke T Schultheis C Gessler M Leimeister C Volff JN Transposable elements as a source of genetic innovation: expression and evolution of a family of retrotransposon-derived neogenes in mammals Gene 2005 345 101 111 10.1016/j.gene.2004.11.022 15716091

[B45] Jurka J Kapitonov VV Kohany O Jurka MV Repetitive sequences in complex genomes: structure and evolution Annu Rev Genomics Hum Genet 2007 8 241 259 10.1146/annurev.genom.8.080706.092416 17506661

[B46] Volff JN Turning junk into gold: domestication of transposable elements and the creation of new genes in eukaryotes Bioessays 2006 28 913 922 10.1002/bies.20452 16937363

[B47] Kazazian HH Jr Mobile elements: drivers of genome evolution Science 2004 303 1626 1632 10.1126/science.1089670 15016989

[B48] Hurst GDD Schilthuizen M Selfish genetic elements and speciation Heredity 1998 80 2 8 10.1046/j.1365-2540.1998.00337.x

[B49] Sonigo P Barker C Hunter E Wain-Hobson S Nucleotide sequence of Mason-Pfizer monkey virus: an immunosuppressive D-type retrovirus Cell 1986 45 375 385 10.1016/0092-8674(86)90323-5 2421920

[B50] Perrin L Kaiser L Yerly S Travel and the spread of HIV-1 genetic variants Lancet Infect Dis 2003 3 22 27 10.1016/S1473-3099(03)00484-5 12505029

[B51] Rambaut A Posada D Crandall KA Holmes EC The causes and consequences of HIV evolution Nat Rev Genet 2004 5 52 61 10.1038/nrg1246 14708016

[B52] Berkhout B Gorelick R Summers MF Mely Y Darlix J 6th International Symposium on Retroviral Nucleocapsid Retrovirology 2008 5 21 10.1186/1742-4690-5-21 18298807 PMC2276516

[B53] Cascella M Micheletti C Rothlisberger U Carloni P Evolutionarily conserved functional mechanics across pepsin-like and retroviral aspartic proteases J Am Chem Soc 2005 127 3734 3742 10.1021/ja044608+ 15771507

[B54] Hornak V Okur A Rizzo RC Simmerling C HIV-1 protease flaps spontaneously close to the correct structure in simulations following manual placement of an inhibitor into the open state J Am Chem Soc 2006 128 2812 2813 10.1021/ja058211x 16506755 PMC2555982

[B55] Wright DA Townsend JA Winfrey RJ Jr Irwin PA Rajagopal J Lonosky M Hall BD Jondle MD Voytas DF High-frequency homologous recombination in plants mediated by zinc-finger nucleases Plant J 2005 44 693 705 10.1111/j.1365-313X.2005.02551.x 16262717

[B56] Singleton TL Levin HL A long terminal repeat retrotransposon of fission yeast has strong preferences for specific sites of insertion Eukaryot Cell 2002 1 44 55 10.1128/EC.01.1.44-55.2002 12455970 PMC118054

[B57] Wilke CO Quasispecies theory in the context of population genetics BMC Evolutionary Biology 2005 5 44 10.1186/1471-2148-5-44 16107214 PMC1208876

[B58] National Center of Biotechnology Information http://www.ncbi.nlm.nih.gov

[B59] Gag-pol tree http://gydb.uv.es/gydb/phylogeny.php?tree=gagpol

[B60] Llorens C Futami R Moya A The GyDB collection: Ty3/Gypsy and Retroviridae LTR retroelements and related nonviral proteins Biotechvana Bioinformatics 2008 CR: GyDB Collection

[B61] Gag-pol multiple alignment URL http://gydb.uv.es/biotechvana/collection/alignment.php?alignment=GAGPOL_retroelement&format=htm

[B62] Genedoc http://www.nrbsc.org/gfx/genedoc/index.html

[B63] Llorens C Futami R Vicente-Ripolles M Moya A The CheckAlign logo-maker application in analyses of both gapped and ungapped DNA and protein alignments Biotechvana Bioinformatics 2008 SOFT: CheckAlign

[B64] Shannon CE The mathematical theory of communication. 1963 MD Comput 1997 14 306 317 9230594

[B65] Schneider TD Stephens RM Sequence Logos – A New Way to Display Consensus Sequences Nucleic Acids Research 1990 18 6097 6100 10.1093/nar/18.20.6097 2172928 PMC332411

[B66] Schneider TD Stormo GD Gold L Ehrenfeucht A Information content of binding sites on nucleotide sequences J Mol Biol 1986 188 415 431 10.1016/0022-2836(86)90165-8 3525846

[B67] Schneider TD Consensus sequence Zen Appl Bioinformatics 2002 1 111 119 15130839 PMC1852464

[B68] Tom Schneider Web site http://www-lecb.ncifcrf.gov/~toms/

[B69] Louis JM Dyda F Nashed NT Kimmel AR Davies DR Hydrophilic peptides derived from the transframe region of Gag-Pol inhibit the HIV-1 protease Biochemistry 1998 37 2105 2110 10.1021/bi972059x 9485357

[B70] Schwede T Kopp J Guex N Peitsch MC SWISS-MODEL: An automated protein homology-modeling server Nucleic Acids Res 2003 31 3381 3385 10.1093/nar/gkg520 12824332 PMC168927

[B71] RCSB Protein Data Bank http://www.rcsb.org/pdb/home/home.do

[B72] PHYLIP package of programs for inferring phylogenies. Version 3.6a3 http://evolution.genetics.washington.edu/phylip.html

[B73] Eck RV Dayhoff MO Atlas of Protein Sequence and Structure 1966 National Biomedical Research Foundation, Silver Spring, Maryland

[B74] Fitch WM Toward Defining the Course of Evolution: Minimum Change for a Specific Tree Topology Systematic Zoology 1971 20 406 416 10.2307/2412116

[B75] Margus T McMorris FR Consensus n-trees Bull Math Biol 1981 43 239 244

[B76] Saitou N Nei M The neighbor-joining method: a new method for reconstructing phylogenetic trees Mol Biol Evol 1987 4 406 425 3447015 10.1093/oxfordjournals.molbev.a040454

[B77] Miyamoto MM Cracraft JL Phylogenetic inference, DNA sequence analysis, and the future of molecular systematics 1991 Oxford University Press, Oxford, England

[B78] Nei M Kumar S Molecular evolution and phylogenetics 2000 Oxford University Press, Oxford, England

