# Supplementary figures and images for: Relationships of gag-pol diversity between Ty3/Gypsy and Retroviridae LTR retroelements and the three kings hypothesis
Source: BMC Evol Biol. 2008 Oct 8;8:276. doi: 10.1186/1471-2148-8-276 (PMC2577118; doi:10.1186/1471-2148-8-276)

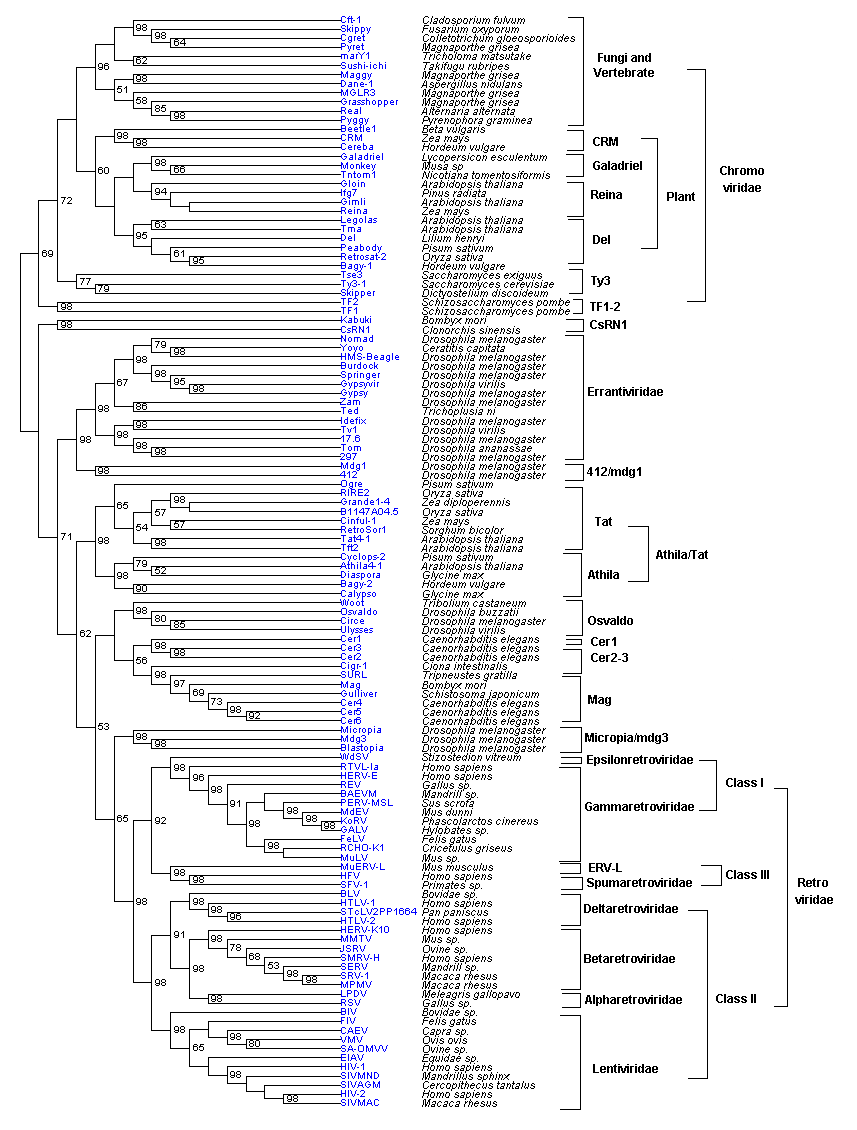

Supplement: Additional File 1 — Expanding gag-pol phylogeny. Expanded version of gag-pol tree illustrated in Figure 1 inferred based on the 120 Ty3/Gypsy and Retroviridae LTR retroelements used in this study. The tree includes information about the names, Genbank accessions and hosts of all LTR retroelement taxa used. By clicking the name of each OTU, the user can locate a file at GyDB providing information of the sequence selected, including a link to its Genbank accession at NCBI. [file 1471-2148-8-276-S1.zip › additional_file_1/gagpol.png]
